# Inconclusive decisions and error rates in forensic science

**DOI:** 10.1016/j.fsisyn.2024.100472

**Published:** 2024-05-04

**Authors:** H. Swofford, S. Lund, H. Iyer, J. Butler, J. Soons, R. Thompson, V. Desiderio, J.P. Jones, R. Ramotowski

**Affiliations:** National Institute of Standards and Technology (NIST), USA

**Keywords:** Forensic science, Error rates, Inconclusives, Likelihood ratio, Validation data, Bayesian reasoning, Black-box study

## Abstract

In recent years, there has been discussion and controversy relating to the treatment of inconclusive decisions in forensic feature comparison disciplines when considering the reliability of examination methods and results. In this article, we offer a brief review of the various viewpoints and suggestions that have been recently put forth, followed by a solution that we believe addresses the treatment of inconclusive decisions. We consider the issues in the context of *method conformance* and *method performance* as two distinct concepts, both of which are necessary for the determination of reliability. Method conformance relates to an assessment of whether the outcome of a method is the result of the analyst's adherence to the procedures that define the method. Method performance reflects the capacity of a method to discriminate between different propositions of interest (e.g., mated and non-mated comparisons). We then discuss implications of these issues for the forensic science community.

## Highlights

• Determination of reliability requires consideration of both method conformance and method performance.
• Error rates alone do not adequately characterize method performance for non-binary conclusion scales.
• Inconclusive decisions are neither “correct” nor “incorrect” but can be either “appropriate” or “inappropriate.”
• Studies that characterize the performance of a particular method are only relevant if conformance can be demonstrated.

## Disclaimers

These opinions, recommendations, findings, and conclusions do not necessarily reflect the views or policies of NIST or the United States Government.

One or more of the authors of this paper serve(s) as an Associate Editor/the Editor-in-Chief of this journal. The standard peer review process was followed and an editor who is not on the author panel has handled the review process for this paper. The authors had no influence over the peer review process. The final decision is made by an editor who is not on the author panel.

## 1 Introduction

The forensic science community faces scrutiny from legal and scientific scholars, who question (measures for) the reliability^1^ of forensic examination methods, with particular emphasis on those that rely predominantly on visual observation and human judgment (e.g., feature comparison methods used in pattern evidence examination, such as friction ridge, firearms and toolmarks, footwear, tire tracks, handwriting) [1,2]. In the 1993 Supreme Court ruling in *Daubert v. Merrell Dow Pharmaceuticals, Inc*. [3], the Court declared that scientific evidence must be relevant and reliable, and provided examples of factors to consider when evaluating its admissibility, such as testability, peer review, error rates, standards, and acceptance in the scientific community. Largely in response to *Daubert*, error rates (e.g., false positive or false negative rates) began to receive increased attention as a key measure of performance.

In 2009, the National Research Council (NRC) report on forensic science renewed the call for determinations of error rates [1] and set in motion efforts to design and execute large-scale testing schemes to evaluate reliability across forensic science disciplines, with an initial emphasis on friction ridge and firearms analyses [[4], [5], [6], [7], [8], [9], [10]]. Likewise, the 2016 report by the President's Council of Advisors on Science and Technology (PCAST) emphasized the need for empirical measures of performance and appropriate determinations of error rates as factors underlying determinations of validity and reliability [2].

The focus on error rates as a primary measure of method performance is generally satisfactory when experts report results using a binary scale, such as identification or exclusion. In this context, the false positive rate is defined as the proportion of times the method results in an “identification” in non-mated comparisons (e.g., in a validation study) and the false negative rate is defined as the proportion of times the method results in an “exclusion” in mated comparisons.^2^ However, few feature comparison disciplines operate using a binary scale. Most use a three-point (or more) scale, which is some variation of identification, inconclusive, or exclusion.^3^ Even with the additional option of inconclusive, it might seem natural to apply the classical definitions of false positive rate and false negative rate. However, careful consideration quickly reveals that it is unsatisfactory to use error rates alone as the metric of performance for a method in these feature comparison disciplines.

Consider the following hyperbolic example to illustrate this point (Table 1a, Table 1ba and 1b).^4^ Suppose we have two methods with the following outcomes for mated and non-mated comparisons.

**Table 1a:** A 2 × 3 table representing performance metrics relating to hypothetical Method 1 where all reported outcomes for both mated and non-mated comparisons are “inconclusive.”

Method 1	Identification	Inconclusive	Exclusion
Mated Comparisons	0 %	100 %	0 %
Non-Mated Comparisons	0 %	100 %	0 %

**Table 1b:** A 2 × 3 table representing performance metrics relating to hypothetical Method 2 where all reported outcomes for mated comparisons are “Identification” and all non-mated comparisons are “Exclusion.”

Method 2	Identification	Inconclusive	Exclusion
Mated Comparisons	100 %	0 %	0 %
Non-Mated Comparisons	0 %	0 %	100 %

In Table 1a, Table 1ba and 1b, we see that neither Method 1 nor Method 2 results in any identification decisions for non-mated comparisons or exclusion decisions for mated comparisons. Therefore, both methods have the ideal false positive and false negative rates of 0 % (or correspondingly, a seemingly ideal total combined error rate of 0 %). The usefulness of the two methods, however, could not be further apart.

The purpose of the forensic examination (e.g., in feature comparison disciplines) is to help others determine whether or not two patterns could have originated from the same source. Thus, a method's utility is characterized by how successfully the method's output distinguishes non-mated comparisons from mated comparisons. Method 1 leads to an inconclusive result for every comparison. This outcome means that Method 1 does not provide any information to help a user of the reported result (e.g., factfinder) determine whether a given comparison is non-mated or mated. Method 2, however, perfectly distinguishes all non-mated comparisons from mated comparisons. That is, a user of the reported result who inferred a comparison was non-mated if the result from Method 2 was exclusion and inferred a comparison was mated if the result was identification would have been correct every time. This example illustrates that, when a conclusion scale is not binary, false positive and false negative rates alone do not accurately convey how successfully one could use the method output to distinguish non-mated comparisons from mated comparisons and therefore do not adequately characterize method performance.^5^

Nevertheless, perhaps motivated by the fact that the term “error rates” is explicitly mentioned in the *Daubert* decision as well as the NRC and PCAST reports, the desire to represent method performance in terms of error rates has continued. Consequently, disagreements over the treatment of inconclusive decisions also remain. To avoid the misleading nature of classical definitions for false positive rate and false negative rate for non-binary conclusion scales, alternative definitions for false positive and false negative rates have been proposed—primarily manifesting in various ways of treating inconclusive outcomes. For example, the PCAST suggested omitting inconclusive decisions altogether so that (error) rate estimates are based on the proportion of conclusive examinations rather than the proportion of all examinations [2].

Although PCAST touched on this issue in 2016, controversy surrounding the treatment of inconclusive decisions began to surface in 2019 when Dror and Langenburg raised concern that there is a lack of transparency and accountability on the use of inconclusive decisions and recommended that the forensic science community establish criteria to know whether and when inconclusive decisions are “justifiable” [11]. This was followed by recommendations by Dror and Scurich in 2020 in which inconclusive decisions that did not conform to some established criteria ought to be counted as errors [12]. Not long after, several different articles were published expressing various viewpoints relating to the treatment of inconclusive decisions [[13], [14], [15], [16], [17], [18]].

When deliberating on this issue, nearly every possible option has been proposed, including: inconclusive decisions be ignored altogether, inconclusive decisions always be considered correct, inconclusive decisions always be considered incorrect, inconclusive decisions be considered correct in some situations and incorrect in other situations, and inconclusive decisions be considered neither correct nor incorrect. Consequently, we are left with an array of proposed definitions of false positive and false negative rates that can lead to wildly different estimates of error rates, and, therefore, different representations and interpretations of the reliability of forensic science results, all with potential consequences regarding the admissibility of such evidence in judicial proceedings.

## 2 Discussion

When considering how inconclusive decisions should be treated (or any outcome for that matter), it is important to first take a step back and frame the context of the situation. There are two important things to consider:

First, in forensic casework, a particular issue might be disputed and the ground-truth of that issue (e.g., true source-origin of a particular set of compared items) is unknown and, oftentimes, unknowable. Further, items or impressions from crime scenes are often presented to analysts in a partial, degraded, or low-quality state. Thus, it is certainly conceivable that forensic analysts will encounter situations where an examination does not yield sufficient information to support a conclusive opinion as to the potential source. Thus, an inconclusive determination is a possible, and sometimes necessary and important, outcome of the examination to ensure a binary decision (e.g., exclusion or identification) is not forced where it is not warranted and achievable. We recognize that this point is largely uncontroversial. What is contentious, however, is when inconclusive determinations might be warranted or justifiable and how inconclusive determinations should be treated when assessing the reliability of a method.

Second, users of forensic results (e.g., factfinders) are presented with the outcome of an examination conducted by a particular analyst and tasked with making inferences and decisions about the truth of various propositions in question (e.g., whether or not two patterns originated from the same source). Users of the reported result must therefore weigh the reliability of the result by considering at least three questions.

(1) What method did the analyst apply when conducting the forensic examination?
(2) How effective is that method at discriminating between the propositions of interest?
(3) How relevant is the data describing the discriminability (i.e., diagnostic capacity) of that method (generally) to the examination in the case at hand (specifically)?

To address these questions, information about whether the analyst conformed to a particular method as well as measures relating to the performance of that method are needed. In this context, we distinguish between two important concepts: *method conformance* and *method performance*.

• Method conformance relates to assessments of whether the outcome of a particular method is the result of the analyst's adherence to the procedures that define that method.
• Method performance relates to measures that reflect the extent to which the outcome of a particular method can effectively distinguish between different propositions of interest (e.g., between same-source and different-source comparisons).

Method performance includes information relating to both *discriminability* and *reproducibility* of outcomes produced by the method.^6^ Importantly, measures of reproducibility provide the gauge by which measures of discriminability (based on outcomes from multiple analysts generally) are relevant to an outcome by a particular analyst (specifically) as well as the adequacy of the procedures that define the method.^7^ Further, while measures of method performance are the means by which methods are deemed “acceptable” for the intended application (e.g., from a validation study),^8^ those measures of performance are only applicable to the extent that assessments of conformance are possible. Thus, determinations of reliability require consideration of results in the context of both method conformance *and* method performance.

In reviewing previously published viewpoints, we see several attempts to provide a better way of assessing the reliability of analysts’ decisions. However, there are three general issues that we consider to have caused many of these prior viewpoints to be incomplete (1) error rates alone (i.e., false positive and false negative rates): have been used as primary measures of method performance despite being unsuitable for non-binary conclusion frameworks, (2) measures of reproducibility (or other factors that do not consider decision outcomes in relation to ground-truth) have been conflated with measures of discriminability, and (3) assessments of method conformance have not been fully considered as a necessary factor for determinations of reliability for a particular case. A brief description of the viewpoints from eight different articles is provided in Table 2. A summary assessment of each article and a more detailed discussion of these three issues follows.^9^

**Table 2:** Brief description of recent articles discussing the treatment of inconclusive decisions in forensic science.

	Articles	Description of Viewpoints
1	*Dror and Langenburg (2019)* [11]	Called for greater transparency and accountability for the use of inconclusive decisions. An option of inconclusive should not be available when there is sufficient information to make a conclusive decision to avoid an “easy way out.” They supported developing criteria to determine situations where fingerprint examiners would not be allowed to choose inconclusive and to use statistical models or qualified opinion scales that provide greater distinction of the perceived strength of evidence within the broad inconclusive category along with blind verification to assess appropriateness of an inconclusive decision.
2	*Dror and Scurich (2020)* [12]	Recognized the need for inconclusive decisions in some cases but claimed that these decisions ought to be considered correct or incorrect based on whether the evidence contains sufficient quantity and quality of information for a conclusive determination. They proposed either using a panel of independent experts or consensus data from a study to determine which comparisons should be deemed as inconclusive.
3	*Weller and Morris (2020)* [13]	Suggested that the rates of all decision types be reported as they relate to ground-truth with the recognition that there are two ground-truth states and three meaningful response categories. They expressed concerns with Dror and Scurich (2020) views of categorizing every result as correct or erroneous and representing measures of reproducibility as measures of accuracy.
4	*Hofmann* et al. *(2020)* [14]	Outlined and critiqued four approaches to address inconclusive decisions in calculating error rates, such that inconclusive decisions are: (1) ignored altogether, (2) considered as correct, (3) considered as incorrect, and (4) considered equivalent to an exclusion. They distinguished between “source-specific” and “decision-specific” metrics, suggesting they should be used for different purposes (method performance and court testimony).
5	*Biedermann and Kotsoglou (2021)* [15]	Argued that Dror and Scurich (2020) views conflate the ontological level of analysis (where ground-truth is fixed) with the epistemic level of analysis (where ground-truth remains uncertain). They warned against the artificial category of a “forensically correct” determination that does not have a ground-truth. They encouraged monitoring all response types as they relate to ground-truth so that the true limits of the method can be understood.
6	*Arkes and Koehler (2021)* [16]	Emphasized that inconclusive decisions are a statement about the insufficiency of available evidence and are neither correct nor incorrect as there is no applicable ground-truth. They proposed the use of signal detection theory as a framework for understanding the role inconclusive decisions play and opposed scoring inconclusives as either correct or incorrect when computing error rates.
7	*Dorfman and Valliant (2022)* [17]	Described an ideal “mechanical scheme” for establishing an objective basis to categorize inconclusive decisions as errors using objective measurements, statistical algorithms, and likelihood theory and illustrated how this could be used to assess overall error rates as described by Dror and Scurich (2020). Until such measures are available, they suggested blind testing schemes be employed to estimate error rates and that inconclusive decisions must be regarded as potential errors.
8	*Guyll* et al. *(2023)* [18]	Argued that inconclusive decisions are different because they forgo any assertion as to the ground-truth state of the evidence. They advocated for the rates of all decision types to be reported as they relate to ground-truth, conclusive and inconclusive alike, to make results useful for the widest range of purposes. They also suggested that the likelihood ratio of a decision (e.g., calculated in terms of “the proportion of all same-source comparisons that are given a particular decision divided by the proportion of all different-source comparisons that are given that same decision”) be used as a metric for expressing its “probative value.” They recognized, however, that evaluations of a technique for designating “decision correctness” (such as the use of a decision rule, consensus opinion, or similarity measure with cutoff criterion) may be useful in some contexts, such as training or determining appropriateness of examiners' decision in relation to evidence quality.

Dror and Langenburg (2019) [11], Dror and Scurich (2020) [12], Hofmann et al. (2020) [14], and Dorfman and Valliant (2022) [17] focused predominantly on the use of error rates as primary measures of performance. In doing so, they offered multiple alternative definitions of error rates through different treatments of inconclusive responses. These alternative definitions conflate (explicitly or implicitly) measures of reproducibility (or other factors that do not consider decision outcomes in relation to ground truth) with measures of discriminability (i.e., suggesting that analysts' decisions that are not consistent with majority or expert panels, or do not conform to method-specific decision criteria, can be represented as erroneous outcomes). The decision-specific metrics discussed by Hofmann et al. [14] are affected by the prior odds of mated versus non-mated samples. For a performance study, this is determined by the arbitrary choice of the ratio of the respective comparisons. For court testimony, the evaluation of prior odds is typically outside the purview of the forensic evaluation. Thus, such decision-specific metrics do not provide clear information regarding a method's ability to discriminate between the propositions of interest. Arkes and Koehler (2021) [16] seemed to implicitly perpetuate the use of error rates as primary measures of performance. They did, however, touch on the concept of method conformance as distinct from method performance. Weller and Morris (2020) [13], Biedermann and Kotsoglou (2021) [15], and Guyll et al. (2023) [18] recognized the misleading and incomplete nature of error rates when used as sole measures of method performance for non-binary conclusion scales and instead advocated for presenting all decision outcomes when representing performance. Guyll et al. [18] touched on the concept of method conformance as distinct from method performance. However, framing conformance considerations as “decision correctness” conflates the concepts and may cause confusion. Guyll et al. [18] went further and proposed an alternative non-error rate metric—a likelihood ratio for each possible result—that can help convey how successfully one could use the method output to distinguish non-mated comparisons from mated comparisons.

### 2.1 Issue 1: focusing solely on two (error) rates

The first issue of concern is the focus on two (error) rates to represent method performance for non-binary conclusion scales. This approach overlooks important details about the performance of the method, and the array of proposals for different ways of computing false positive and false negative rates could be seen as a discussion of which details should be overlooked. That is, using two error rates as a sole measure of performance loses information relative to presenting the rate of each decision level (e.g., exclusion, inconclusive, identification) for non-mated comparisons and for mated comparisons (e.g., a 2 × 3 table, representing the two ground-truth states and three possible decision outcomes, as illustrated by Table 1a, Table 1ba and 1b). This is evident by noting that, regardless of what definitions are adopted for false positive rate and false negative rate, the full 2 × 3 table is not recoverable from these two numbers. For each of the proposed approaches for computing error rates, examples can be readily constructed of two methods that produce identical error rates but have different abilities to discriminate non-mated comparisons from mated comparisons or have different levels of reproducibility. Thus, for non-binary conclusion scales, error rates alone do not provide sufficient information for characterizing method performance (i.e., discriminability and reproducibility). This issue of losing information also extends to other summaries of performance where the full 2 × 3 table is not recoverable, such as the area under the receiver operator characteristic curve (AUC) or empirical cross entropy (ECE) [19].

Additionally, computing error rates raises the question of how to label inconclusive decisions. This has led to the various viewpoints summarized in Table 2 and some controversy because inconclusive decisions are not necessarily correct or incorrect. A “correct” decision is one that accurately represents the true source-origin state of items being compared. An “incorrect” decision is one that falsely represents the true source-origin state, resulting in an error (i.e., falsely asserting that two impressions originated from the same source or falsely asserting that two impressions originated from different sources). An inconclusive decision, on the other hand, is an outcome of the examination for which an assertion about the source-origin state of the items being compared was not explicitly made. Thus, an inconclusive decision is neither a correct nor erroneous representation of the true source-origin state. Other summaries, such as AUC or ECE offer an advantage in the sense that they do not require such binary labels; however, any summary from which the 2 × 3 table cannot be reconstructed is unsuitable for providing a complete characterization of a method's performance in discriminating between the propositions of interest.

Information regarding method performance should help others assess what weight to give to the method's result in a given case (for which ground-truth is not known). For instance, as noted by Guyll et al. [18], one could consider the “probative value” of the result by assessing the likelihood ratio for the analyst's decision using data collected under relevant conditions (e.g., approximated by calculating the portion of all mated comparisons for a particular decision divided by the portion of all non-mated comparisons for the same decision). This requires a complete and transparent representation of all possible outcomes as they relate to ground-truth of the compared items under specified conditions. Thus, when considering a more suitable way of conveying performance characteristics, we agree with the viewpoints and suggestions put forth by Weller and Morris [13], Biedermann and Kotsoglou [15], and Guyll et al. [18]—to provide the entire table of outputs representing all possible outcomes (e.g., a 2 × 3 table, such as that represented in Table 1a, Table 1b, Table 3a, Table 3bb).^10^ This provides greater transparency about the method's performance and enables users of the information to more effectively discriminate between propositions of interest (i.e., mated versus non-mated).

**Table 3a:** A 2 × 3 table representing performance metrics relating to hypothetical Method 3.

Method 3	Identification	Inconclusive	Exclusion
Mated Comparisons	89 %	10 %	1 %
Non-Mated Comparisons	1 %	40 %	59 %

**Table 3b:** A 2 × 3 table representing performance metrics relating to hypothetical Method 4.

Method 4	Identification	Inconclusive	Exclusion
Mated Comparisons	59 %	40 %	1 %
Non-Mated Comparisons	1 %	10 %	89 %

Consider the following 2 × 3 tables describing results of validation testing from hypothetical methods 3 and 4, reflected in Table 3a, Table 3ba and 3b.

There are several performance summaries for which methods 3 and 4 appear equivalent (e.g., error rates, AUC).^11^ However, the complete tables reveal several important differences between the methods. Table 3a indicates that inconclusive decisions from method 3 occur at a rate among non-mated comparisons that is four times greater than the rate among mated comparisons. Table 3b, however, indicates that inconclusive decisions from method 4 occur at a rate among mated comparisons that is four times greater than the rate among non-mated comparisons. Thus, inconclusive decisions have different implications depending on whether they resulted from method 3 or method 4. The implied “probative value” of inconclusive decisions between methods 3 and 4 differ by a factor of 16. Differences also occur for identification and exclusion decisions. Decisions made by factfinders (or others within the criminal justice system, such as investigators, litigators, or judges) in response to an expert's opinion in a given case may depend on whether the expert applied method 3 or 4 (i.e., they may make different decisions depending on whether Table 3a or 3b is provided). This example illustrates the general fact that any summary of method performance from which the 2 × 3 table cannot be inferred risks losing information important for assessing what weight to give an expert's opinion in a given case.

Presenting the complete 2 × 3 table ensures that users of the information can make the best possible decision for the relevant conditions in the case. This is particularly true when inconclusive decisions are not symmetrically distributed between mated and non-mated comparisons. Excluding inconclusive decisions, combining them into a different category of decisions (for purposes of labeling them as correct or incorrect decisions),^12^ or only representing incomplete summary statistics reflecting a subset of performance characteristics of the method (such that the 2 × 3 table cannot be reconstructed) prevents a meaningful interpretation of the performance of the method. Instead, such treatment of inconclusive decisions causes those performance characteristics to be represented in a distorted and potentially misleading way that can ultimately lead to fewer accurate factfinder decisions overall. Appendix I discusses this in more detail based on two pillars of statistical inference dealing with optimal decision making—Bayesian decision theory [20,21] and the Neyman-Pearson Lemma [22].

### 2.2 Issue 2: conflating reproducibility with discriminability

The second issue of concern is the suggestion that measures of reproducibility can be used as the basis for representing measures of discriminability of the method. Measures of reproducibility do not consider decision outcomes in relation to ground-truth; thus, they cannot provide a complete representation of the accuracy of an outcome or a method's utility in discriminating between non-mated and mated comparisons. At most, they provide limited information regarding discriminability (i.e., imperfect reproducibility indicates imperfect accuracy).

One approach to represent reproducibility data for a three-point conclusion scale is through a 3 × 3 table (e.g., Table 4). The data reflected in 3 × 3 tables provide an indication of the adequacy of the procedures that define the method. A 3 × 3 table formed using outcomes that have been assessed as properly conforming to the procedures that define a particular method reflects the extent to which the method can produce consistent results and the variability between laboratories or analysts for a given input and conditions. To the extent that measures of reproducibility among such decisions (i.e., variability among laboratories or analysts) are acceptable, the procedures that define the method and approaches for assessing conformance are adequate (i.e., the method is sufficiently well-defined and conformance to those procedures can be effectively demonstrated). However, if the measures of reproducibility among such decisions are such that it is common for different analysts to reach different decisions for a given input and conditions, or if the extent of the variability is otherwise unacceptable, then the procedures that define the method might be not be adequately specified (i.e., loosely defined) or the approaches for assessing conformance might not be sufficient (i.e., outcomes have been improperly assessed as conforming).

**Table 4:**
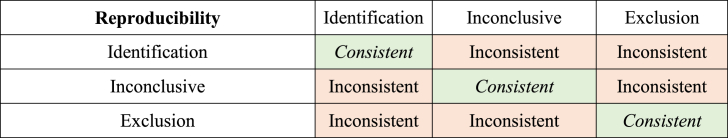
An example 3 × 3 table representing the reproducibility of decisions for a method. The table reflects the extent to which multiple applications of the same method between different laboratories or analysts produce consistent results. A well-defined method will yield a high proportion of consistent outcomes. Inconsistent outcomes reflect the extent of variability between laboratories or analysts and any ambiguity on what the method can be expected to produce for a given input and conditions.

The data reflected in 3 × 3 tables also provide an indication of the extent to which aggregate measures of discriminability (reflected by a 2 × 3 table) across multiple analysts for a given method are relevant to a particular analyst's application of that method. While high measures of reproducibility indicate that analysts are performing with similar levels of discriminability, this is not necessarily true when measures of reproducibility are lower. Although lower measures of reproducibility will have some impact on aggregate measures of discriminability, it might not be clear whether that impact is due to some analysts performing poorly and other analysts performing well or due to all analysts performing mediocre. In other words, when measures of reproducibility are low, there could be substantial differences between assessments of performance based on the pooled 2 × 3 discrimination table and the corresponding table constructed using data for any given individual analyst. In that case, when presented with an outcome from a particular analyst for whom individual performance data is not available (as is often the case in practice), there will be no way to know where that analyst aligns in terms of the full range of performance among other analysts represented by the aggregate performance data. Thus, aggregate measures of reproducibility provide a gauge by which measures of discriminability (based on outcomes from multiple analysts generally) are relevant to an outcome by a particular analyst (specifically).

Measures of reproducibility (e.g., as reflected in 3 × 3 tables) can be obtained without knowing the ground-truth state (i.e., whether the comparisons are mated or non-mated), and can therefore be evaluated from actual casework data, at least conceptually. While these tables provide useful information, no summary from a 3 × 3 reproducibility table can provide the essential information contained in a 2 × 3 discrimination table, such as those illustrated in Table 1a, Table 1b, Table 3a, Table 3bb. The diagonal and off-diagonal elements of the 3 × 3 tables (labeled as “consistent” and “inconsistent” outcomes, respectively, in Table 4) are measures of (ir)reproducibility and must not be mistaken as suitable summaries of method discrimination.

This issue with using measures of reproducibility as a means of representing measures of discriminability also extends to the use of any other criteria or factors that do not consider results in relation to ground-truth (e.g., based on assessments of method conformance or comparing outcomes from one method to those from another method).^13^

### 2.3 Issue 3: lack of considerations for method conformance

The third issue of concern is the limited appreciation for the importance of method conformance when assessing or reporting measures of method performance. Method conformance is related to method performance. Performance data for one method is not relevant to a different method. If an analyst deviates from procedures for a particular comparison, they are no longer using the method specified by those procedures. Deviating from the procedures does not mean that an analyst is necessarily performing better or worse than those analysts following the procedures; however, it does mean that performance data for that method (i.e., from the other analysts who did follow the procedures, such as assessed during validation studies) might not adequately reflect the performance of the given analyst for the comparison in question, which could leave little or no information with which to assess the reliability of the outcome produced by the non-conforming analyst.

### 2.4 Evaluation of results

Taking into consideration these three issues, in the context of measuring *method performance*, we stress that the discriminability of analysts' decisions can only be assessed in terms of ground-truth, and because “inconclusive” decisions are not an assertion about the source-origin state of the items being compared, they are neither “correct” nor “incorrect.” However, in the context of assessing *method conformance*, all analysts’ decisions (including inconclusive decisions) should be assessed as “appropriate” or “inappropriate” in terms of whether they resulted from a proper application of a specified method. Thus, we agree with Dror and Langenburg [11] and Dror and Scurich [12], in the sense that one might wish to assess whether a particular decision, such as an inconclusive, is “justifiable.” Whether a particular decision is “justifiable,” however, depends on whether the outcome of the examination was “appropriate” (i.e., produced by proper conformance to the method procedures, including relevant decision criteria, if applicable) and whether empirical measures relating to the performance of that method (i.e., discriminability and reproducibility) under conditions relevant to a particular case have been deemed “acceptable.” A result that is inappropriate does not mean it is incorrect; however, it does mean that there is likely little to no data with which the weight of the result can be assessed.

Consider the following two scenarios, for example, to elaborate on this point using a hypothetical method that includes explicit criteria to support decisions of identification or exclusion (e.g., specified minimum quality and quantity of corresponding or discordant features) and for which performance characteristics of the method have been deemed “acceptable” for use:

(1) When the criteria specified by a method to support a decision of identification or exclusion *have not been met:*
  a. Inconclusive decisions that are produced under this situation represent an outcome that is expected when procedures that define the method are adhered to. Such decisions reflect that the method has been applied in accordance with the scope of its validation and in a manner deemed acceptable for use. Therefore, in this situation, such decisions are *appropriate* as they relate to assessments of method conformance. Of course, the more often a method produces inconclusive outcomes, the less useful it would be and less likely the method might be deemed “acceptable” for operational use.
  b. Identification or exclusion decisions that are produced under this situation represent an outcome that is not expected when the procedures that define the method are adhered to. Such decisions reflect that the method has not been applied in accordance with the scope of its validation of what has been deemed to be acceptable. Therefore, in this situation, such decisions are *inappropriate* as they relate to assessments of method conformance. It is important to note that even if such decisions happen to be correct (based on ground-truth), they still represent an outcome that is not in conformance with the specified requirements, or criteria, deemed to be appropriate and acceptable for the intended use (i.e., the risk and consequences of producing errors when such conclusive decisions are made for a given input and conditions have been deemed to be too great).
(2) When the criteria specified by a method to support a decision of identification or exclusion *have been met:*
  a. Inconclusive decisions that are produced under this situation represent an outcome that is not expected when the procedures that define the method are adhered to. Such decisions reflect that the method has not been applied in accordance with the scope of its validation or in a manner deemed acceptable for use. Therefore, in this situation, such decisions are *inappropriate* as they relate to assessments of method conformance.
  b. Identification or exclusion decisions meeting the relevant criteria that are produced under this situation represent an outcome that is expected when the procedures that define the method are adhered to. Such decisions reflect that the method has been applied in accordance with the scope of its validation of what has been deemed to be acceptable. Therefore, in this situation, such decisions (identification or exclusion, depending on the criteria relevant for each type of conclusive decision) are *appropriate* as they relate to assessments of method conformance. Like the counter-scenario described above (where an outcome might be correct yet inappropriate), it is important to note that even if such conclusive decisions provided under these circumstances happen to be incorrect, they still represent an outcome of the method that is in conformance with the specified requirements, or criteria, deemed to be appropriate and acceptable for the intended use. In other words, although there might be occasions where such decisions are incorrect, the tradeoff between correct and incorrect outcomes has been deemed acceptable to permit use of the method. Of course, the more often a method produces incorrect outcomes, the less useful it would be and less likely the method might be deemed “acceptable” for operational use.

**Fig. 1:**
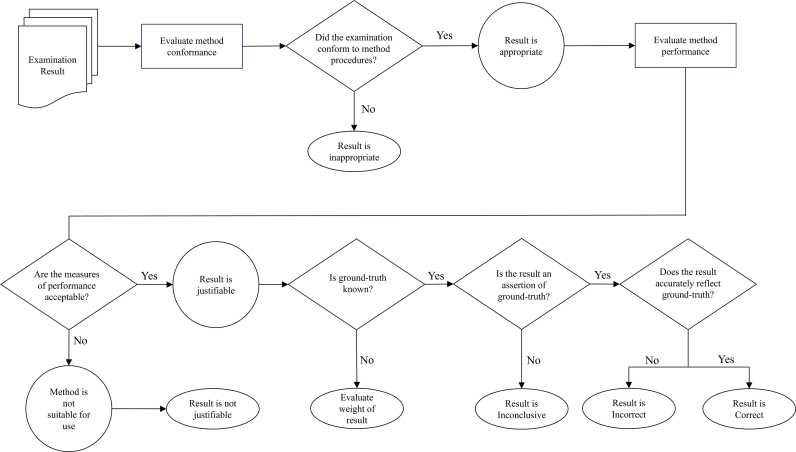
Simplified flow diagram reflecting the process for evaluating examination results. The diagram illustrates the distinctions between results labeled as “appropriate” vs. “inappropriate,” “justifiable” vs. “not justifiable,” and “correct” vs. “incorrect.”

While method conformance and method performance are both important aspects for determinations of reliability, care must be taken not to confuse or conflate the two. These two concepts are distinct, and both must be accounted for separately when considering the reliability of a particular method (e.g., during validation testing) or evaluating the weight of a particular result of a method (e.g., in a particular case). For method conformance, assessments must be based on an empirical demonstration that the established requirements and criteria inherent in the method have been satisfied (e.g., relating to analyses of quality, quantity, similarity, or rarity of comparison features and any relevant and applicable decision criteria).^14^ For method performance, measures of discriminability must be assessed in terms of ground-truth (i.e., mated or non-mated comparisons) and measures of reproducibility must be assessed in terms of the consistency of decisions for a given input and conditions when the same method is applied by different analysts. Importantly, while measures of reproducibility provide an indication of the adequacy of the procedures that define the method (i.e., well-defined procedures produce more consistent results), demonstrating consistency of outcomes (e.g., agreement between analysts) post hoc is not sufficient to serve as a basis for assessing or demonstrating conformance to a method or labeling a result as “appropriate.” Conformance must be assessed and empirically demonstrated based on adherence to procedures that define the method. Once conformance has been demonstrated, performance data for that method can be used to evaluate the weight of an “appropriate” result. Fig. 1 uses a simplified flow diagram to illustrate the process for evaluating examination results and the distinctions between results labeled as “appropriate” vs. “inappropriate,” “justifiable” vs. “not justifiable,” and “correct” vs. “incorrect.”

## 3 Conclusion

Different treatments of inconclusive decisions and calculations of error rates in forensic feature comparison disciplines have led to different representations and interpretations of the reliability of forensic science results. In this paper, we explored these issues in further detail from a metrology perspective and distinguished between the concepts of *method conformance* and *method performance*. We also considered the broader implications of these concepts when determining reliability of analysts’ examination results.

The issues discussed in this paper have several practical implications to researchers and forensic service providers alike. They impact studies and activities relating to method validation and performance monitoring, as well as how results are characterized and communicated—all of which are prescribed by ISO/IEC 17025:2017 [23], the prevailing international standard to which many forensic laboratories conform—and the extent to which performance data are useful for determinations of reliability in casework.^15^ Major implications of these issues and key takeaways from this paper are as follows:

First, determinations of the reliability of analysts’ examination results require consideration of those results in the context of both method conformance *and* method performance—a result alone is not sufficient for one to assess its reliability.

Second, error rates alone do not adequately characterize method performance for non-binary scales. Instead, the entirety of possible outcomes should be provided as it relates to measures of discriminability (i.e., 2 × 3 table) and reproducibility (i.e., 3 × 3 table) constructed from relevant validation testing.

Third, inconclusive decisions are neither “correct” nor “incorrect” (in terms of method performance) but can be either “appropriate” or “inappropriate” (in terms of method conformance).

Fourth, studies that purport to characterize the performance of a *particular* method (i.e., validation studies) are only relevant if conformance to that method can be demonstrated. Therefore, forensic service providers that do not have well documented and detailed step-by-step procedures that define their method, including conditions for method application and decision criteria for results for which performance data can be associated are unlikely to be able to meaningfully support a claim that the outcome of their examination is the product of a reliable method.

Fifth, studies that characterize aggregate measures of performance across a discipline (e.g., black-box studies or interlaboratory comparisons) but do not specify the methods used can provide information about the performance characteristics that can be expected for the practice overall. While these studies are helpful to users of the information, they cannot necessarily serve as a validation or provide generalizable performance characteristics of a *particular* method relevant to a specific case unless it can be shown that the same method was used by all participants. The development and use of standard methods by multiple laboratories is an important step toward reducing variability and ensuring that aggregate measures of performance can be represented as generalized measures of performance for those methods. This standardization, in turn, strengthens the evidence-base^16^ supporting the validation of those methods and reduces the resource burdens that would otherwise be placed on individual laboratories to accomplish these studies independently.

## CRediT authorship contribution statement

**H. Swofford:** Writing – review & editing, Writing – original draft, Conceptualization. **S. Lund:** Writing – review & editing, Writing – original draft, Conceptualization. **H. Iyer:** Writing – review & editing, Writing – original draft, Conceptualization. **J. Butler:** Writing – review & editing, Writing – original draft, Conceptualization. **J. Soons:** Writing – review & editing, Writing – original draft, Conceptualization. **R. Thompson:** Writing – review & editing, Writing – original draft, Conceptualization. **V. Desiderio:** Writing – review & editing, Writing – original draft, Conceptualization. **J.P. Jones:** Writing – review & editing, Writing – original draft, Conceptualization. **R. Ramotowski:** Writing – review & editing, Writing – original draft, Conceptualization.

## Declaration of competing interest

The authors declare that they have no known competing financial interests or personal relationships that could have appeared to influence the work reported in this paper.

## Figures and Tables

**Table AI.1:** A 2 × 3 table of the probabilities for different conclusions an analyst might reach in $H_{p}$-true and $H_{d}$-true scenarios.

Scenario	Identification (ID)	Inconclusive	Exclusion	Total
$H_{p}$-true	P1	P2	P3	100 %
$H_{d}$-true	Q1	Q2	Q3	100 %

**Table AII.1:** A 2 × 3 table representing performance metrics relating to hypothetical Method A.

Method A	Identification (ID)	Inconclusive	Exclusion
Mated Comparisons	59 %	40 %	1 %
Non-Mated Comparisons	1 %	10 %	89 %

**Table AII.2:** A 2 × 3 table representing performance metrics relating to hypothetical Method B.

Method B	Identification (ID)	Inconclusive	Exclusion
Mated Comparisons	40 %	59 %	1 %
Non-Mated Comparisons	10 %	1 %	89 %

## Appendix I

### Explanation for the inadequacy of error rate summaries for factfinder decision making

Ultimately, an expert's opinion is information provided to a factfinder, who is tasked with assessing what weight to give that opinion as part of their decision-making process. Understanding what outcomes have been produced in known ground-truth scenarios (i.e., validation testing) can help factfinders assess the weight of an expert's opinion. Oftentimes, attention centers around the error rates of a given method. However, two pillars of statistical inference dealing with optimal decision making—Bayesian decision theory [1] and the Neyman-Pearson Lemma [2]—show that likelihood ratios, rather than error rates, are the quantities of interest from a 2 × 3 table for factfinders. Computing likelihood ratios requires assessing additional probabilities beyond those that represent error rates. Providing only error rates suppresses information relevant to assessing these additional probabilities. We elaborate on these concepts below.

Consider a factfinder evaluating the prosecution hypothesis $H_{p}$ that the two impressions share the same source, relative to the defense hypothesis $H_{d}$ that they do not. For simplicity, we assume that the factfinder has only two actions available—find the defendant “guilty” or find the defendant “not guilty.” If the factfinder finds the defendant guilty when *H*_*d*_ is true it will lead to a “wrongful conviction.” If the factfinder finds the defendant not guilty when *H*_*p*_ is true, the result will be a “false acquittal.” It is desirable to avoid both situations. Bayesian decision theory provides a principled approach for arriving at an optimal decision strategy and the reader is referred to Ref. [3] for a detailed discussion of how this theory can guide a decision maker in the criminal justice system. In general terms, Bayesian decision theory suggests that, among all available decision strategies, one should choose a decision strategy that minimizes the “expected cost” of the decision.

Assessing the expected cost of a given decision requires the probabilities for various scenarios of interest and the cost the factfinder would associate with errant decisions under each of those scenarios. Suppose the costs the factfinder associates with a wrongful conviction or false acquittal are given by *C*_*wc*_ or *C*_*fa*_, respectively. Suppose the factfinder has a prior probability p for $H_{p}$ (and 1- p for $H_{d}$).^17^ This prior probability reflects the factfinder's state of uncertainty before hearing from the expert and will be updated after learning the result from the forensic analysis.

The process of updating uncertainty in response to new information can be conducted using Bayes’ equation, which requires a likelihood of the new information under each of the scenarios of interest.

Table AI-1 provides the probabilities for the different outcomes an analyst might reach in $H_{p}$-true and $H_{d}$-true scenarios, respectively.^18^ In table AI-1, the value of P1 represents the probability that an expert would provide an “ID” after evaluating a pair of impressions for which $H_{p}$ is true, and the value of Q1 represents the probability that an expert would provide an “ID” after evaluating a pair of impressions for which $H_{d}$ is true.

Let us focus on the situation where the analyst result is “ID”. In this case, the factfinder would like to update their prior probability estimate p in light of the expert's decision. Using Bayes rule, we get:

$$P\left(H_{p}|Expert\;says\;ID\right)=\frac{p∙P1}{p∙P1+\left(1-p\right)∙Q1}=\frac{\frac{p}{1-p}∙\frac{P1}{Q1}}{1+\frac{p}{1-p}∙\frac{P1}{Q1}}$$

We extended equation (1) to illustrate that the evaluation requires the values of P1 and Q1, and includes the ratio of P1/Q1. A factfinder can use their estimated posterior probability to assess their expected cost associated with a decision to convict or to acquit. The expected cost is used to assess whether one decision is better than another—a decision with a lower expected cost is preferred. In this setup, the expected costs for the factfinder's available decisions are:

$${\text{Expected}\;\text{cost}\;\text{of}\;\text{acquittal}=C}_{fa}∙P\left(H_{p}|Expert\;says\;\text{ID}\right)$$

$${\text{Expected}\;\text{cost}\;\text{of}\;\text{conviction}=C}_{wc}∙P\left(H_{d}|Expert\;says\;\text{ID}\right)\text{,}$$

where $C_{fa}$ is the cost of a false acquittal and $C_{wc}$ is the cost of a wrongful conviction. Ultimately, it is only the ratio C = $C_{wc}/C_{fa}$ that matters when comparing expected costs of different decisions. The quantity C represents how many false acquittals the factfinder would exchange to avoid one false conviction. For simplicity, and without loss of generality, it is common to consider relative costs by taking $C_{fa}=1$ and $C_{wc}=C\text{.}$ Thus, we get:

$$\text{Expected}\;\text{cost}\;\text{of}\;\text{acquittal}=P\left(H_{p}|Expert\;says\;\text{ID}\right)$$

$$\text{Expected}\;\text{cost}\;\text{of}\;\text{conviction}=C∙P\left(H_{d}|Expert\;says\;\text{ID}\right)\text{.}$$

To apply the Bayesian decision-making paradigm, which is generally accepted as normative [3], the factfinder simply picks whichever choice has the lower expected cost. Note that equation (1) makes clear that this process depends on the value of P1/Q1. Thus, P1/Q1 is an important component of Bayesian reasoning.

We continue this explanation to provide another theoretical motivation for the importance of P1/Q1. Under the above setup, a factfinder's expected cost of conviction would be lower than their expected cost of acquittal if and only if:

$$C<\frac{P\left(H_{p}|Expert\;says\;ID\right)}{P\left(H_{d}|Expert\;says\;ID\right)}$$

The right-hand side of this expression is the posterior odds. In the case where exactly two propositions are considered, Bayes rule shows this is equal to:

$$\frac{P\left(H_{p}|Expert\;says\;ID\right)}{P\left(H_{d}|Expert\;says\;ID\right)}=\frac{P1}{Q1}∙\frac{p}{1-p}$$

where P1/Q1 represents the likelihood ratio (LR) that links the prior odds to the posterior odds. (A more general form of Bayes rule, in which the LR is replaced with a Bayes factor, applies to situations when more than two propositions are considered.)

With some algebra, this means the factfinder's expected cost of conviction would be lower than their expected cost of acquittal if and only if:

$$\frac{P1}{Q1}>C∙\frac{1-p}{p}=\frac{C}{Prior\;odds\;of\;H_{p}}=\tau$$

This provides a decision rule in the form of: “find the defendant guilty if and only if LR P1/Q1 is bigger than the threshold τ,” where τ indicates the factfinder's threshold for how probative the expert's opinion must be in order for them to decide the defendant is guilty.

According to the Neyman-Pearson Lemma [2], decision rules based on whether or not a LR is greater than a given threshold are optimal in the sense that no other type of decision rule can produce a higher true positive rate for any given false positive rate (i.e., no other rule could produce more just convictions while maintaining a given rate of false convictions).^19^ Implementing this optimal decision rule required the value P1/Q1.

We have shown, under two hallmarks of statistical reasoning, that the ratio P1/Q1 is directly relevant to the factfinder when the expert says “ID.” Similar reasoning shows that the ratio P2/Q2 is important to the factfinder when the expert says “inconclusive,” and the ratio P3/Q3 is important when the expert says “exclusion.” Thus, it is critical that factfinders have access to information that would assist their assessments of these ratios. Summarizing performance using error rates alone (or any other summary from which the 2 × 3 table cannot be reconstructed) deprives the factfinder of information relevant for updating their beliefs.

## Appendix II

Limitation of Empirical Cross Entropy.

Empirical cross entropy (ECE) produces identical curves for tables AII-1 and AII-2 below. See equation 6.4 in Ref. [1]. However, the implied likelihood ratios for an “ID” in tables AII-1 and AII-2 are 59 %/1 % = 59 and 40 %/10 % = 4, respectively. This illustrates that ECE curves do not convey all the relevant information from a 2 × 3 table.

## Footnotes

1 In this paper, the term “reliable” is used as an all-encompassing term that relates to the extent to which a method can be relied upon to produce accurate and consistent results, and includes the concepts of “validity,” “reproducibility,” and “repeatability.”

2 Non-mated comparisons refer to items that were known to have been made by different sources. Mated comparisons refer to items that were known to have been made by the same source.

3 We recognize there are different ways of conducting feature comparisons and communicating results (e.g., probabilistic, categoric). In this paper, we limit our discussion to the use of conclusion scales that include inconclusive as a legitimate response option since it represents traditional practices in many feature comparison disciplines. Further, we recognize conclusion scales vary in terms of the number of response options available (e.g., some might have multiple derivations of inconclusive, levels of support, or options to declare items “not suitable” for comparison). For simplicity, we focus our discussion on a single catch-all class of “inconclusive” response options that indicates a comparison outcome that is not an explicit assertion of the ground-truth state of the compared items (e.g., comparison outcomes other than “identification” or “exclusion”).

4 In this example, we use we use percentages of total response outcomes for mated and non-mated comparisons for illustrative purposes, but, in real studies, actual numbers should be provided to enable estimation of uncertainty.

5 A similar example could be constructed to show that the alternative metrics of sensitivity (true positive rate) and specificity (true negative rate) also do not adequately characterize method performance. Such examples illustrate the perils of trying to summarize the performance of a method with a non-binary range of conclusions with the same number of parameters as a method with a binary range of conclusions. Two additional independent parameters or rates are required to fully characterize method performance for each element added to a binary conclusion scale.

6 The term “discriminability” refers to the extent to which the outcomes of a method can accurately distinguish between non-mated and mated comparisons. The term “reproducibility” refers to the extent to which the outcomes of a method are consistently produced.

7 This is important when analysts vary in their performance and measures of discriminability and reproducibility are based on aggregate outcomes from multiple analysts.

8 The decision by a user or a group of users that a method is acceptable for its intended purpose does not obligate or constrain others (e.g., factfinders) to accept that determination when they are later tasked with evaluating the evidence in the context of a case.

9 We do not claim this to be a comprehensive list. The eight articles presented here illustrate a range of viewpoints on the topic.

10 For feature comparison disciplines, this can be accomplished using a 2 × 3 table or equivalent rate parameters reflecting the occurrence of identification, exclusion, and inconclusive decisions as they relate to ground-truth of the compared items. A 2 × 3 table is used in this discussion; however, this recommendation generalizes to a 2x*k* table, where *k* is the total number of possible outcomes that can be produced by the method, such as feature comparison disciplines that employ a 5-level scale, 7-level scale, 9-level scale, or another similar type of scale.

11 Table 3a, Table 3ba and 3b lead to different ECE curves, which are reflections of each other about the vertical axis. However, permuting the column labels (i.e., identification, inconclusive, exclusion) in any 2 × 3 table will produce an identical ECE curve. This means that ECE curves also omit information relevant to assessing the weight of an expert opinion. See Appendix II for an example.

12 For example, by calculating error rates after combining inconclusive decisions with identification decisions or exclusion decisions (i.e., treating all inconclusive decisions as if they were identification decisions or exclusion decisions), as was briefly discussed by Hofmann et al. [14] and Cuellar et al. (2024) [24]. Cuellar et al. [24] reference the Food and Drug Administration (FDA) Guidance for evaluating diagnostic testing when “equivocal” or “indeterminant” results are obtained [25]. While the FDA Guidance provides a means of representing a bounded range for possible error rates, the FDA recognize “[t]his may or may not be reasonable for [a given] situation” [25]. In the context of forensic science, we do not believe the FDA guidance is applicable or appropriate because it masks the actual outcomes produced by the method when tested, does not provide a complete representation of the performance of the method, and hinders the ability for a factfinder to assess the weight of a particular result.

13 For example, the 3 × 3 table in Fig. 1 by Dror and Scurich [12] reflects outcomes labeled as “correct” or “error” based on whether there is “sufficient information to justify such a decision,” as determined by method-specific decision criteria (e.g., suggested by Dror and Langenburg [11]), consensus opinion or majority outcomes (suggested by Dror and Scurich [12]), or algorithmic assessments (suggested by Dorfman and Valliant [17]).

14 Different approaches for analyzing quality, quantity, similarity, or rarity of comparison features (e.g., subjective versus algorithmic) or decision criteria or thresholds different from those specified by the method can impact performance and therefore reflect deviations from established procedures that define a particular method.

15 When considering these issues, it is important to keep in mind that ISO/IEC 17025:2017 specifies that the term “method” is “considered synonymous with the term ‘measurement procedure’ as defined in ISO/IEC Guide 99” and is referred to as being defined by a *specific* and *detailed* step-by-step procedure, referred to as a standard operating procedure [23,26].

16 The term “evidence-base” refers to empirical data reflecting the performance of the method under varying conditions.
